# Trial-by-trial variability in cortical responses exhibits scaling of spatial correlations predicted from critical dynamics

**DOI:** 10.1016/j.celrep.2024.113762

**Published:** 2024-02-10

**Authors:** Tiago L. Ribeiro, Peter Jendrichovsky, Shan Yu, Daniel A. Martin, Patrick O. Kanold, Dante R. Chialvo, Dietmar Plenz

**Affiliations:** 1 Section on Critical Brain Dynamics, National Institute of Mental Health, National Institutes of Health, Bethesda, MD 20892, USA; 2 Department of Biomedical Engineering, Johns Hopkins University, Baltimore, MD 21205, USA; 3 Kavli Neuroscience Discovery Institute, Johns Hopkins University, Baltimore, MD 21205, USA; 4 Brainnetome Center, Institute of Automation, Chinese Academy of Sciences, Beijing 100190, China; 5 CAS Center for Excellence in Brain Science and Intelligence Technology, Chinese Academy of Sciences, Beijing 100190, China; 6 Center for Complex Systems & Brain Sciences (CEMSC3), Instituto de Ciencias Físicas, (ICIFI) Escuela de Ciencia y Tecnología, Universidad Nacional de San Martín (UNSAM), San Martín 1650 Buenos Aires, Argentina; 7 Consejo Nacional de Investigaciones Científicas y Técnicas (CONICET), Godoy Cruz 2290 Buenos Aires, Argentina; 8 Lead contact

## Abstract

In the mammalian cortex, even simple sensory inputs or movements activate many neurons, with each neuron responding variably to repeated stimuli—a phenomenon known as trial-by-trial variability. Understanding the spatial patterns and dynamics of this variability is challenging. Using cellular 2-photon imaging, we study visual and auditory responses in the primary cortices of awake mice. We focus on how individual neurons’ responses differed from the overall population. We find consistent spatial correlations in these differences that are unique to each trial and linearly scale with the cortical area observed, a characteristic of critical dynamics as confirmed in our neuronal simulations. Using chronic multi-electrode recordings, we observe similar scaling in the prefrontal and premotor cortex of non-human primates during self-initiated and visually cued motor tasks. These results suggest that trial-by-trial variability, rather than being random noise, reflects a critical, fluctuation-dominated state in the cortex, supporting the brain’s efficiency in processing information.

## In brief

Neurons in the mammalian cortex respond differently to repeated stimuli, a phenomenon known as trial-by-trial variability. Ribeiro et al. show that each trial’s neuronal variability differs from noise and follows a linear growth in correlation length, indicating a critical state of the cortex that optimizes information processing.

## Graphical Abstract

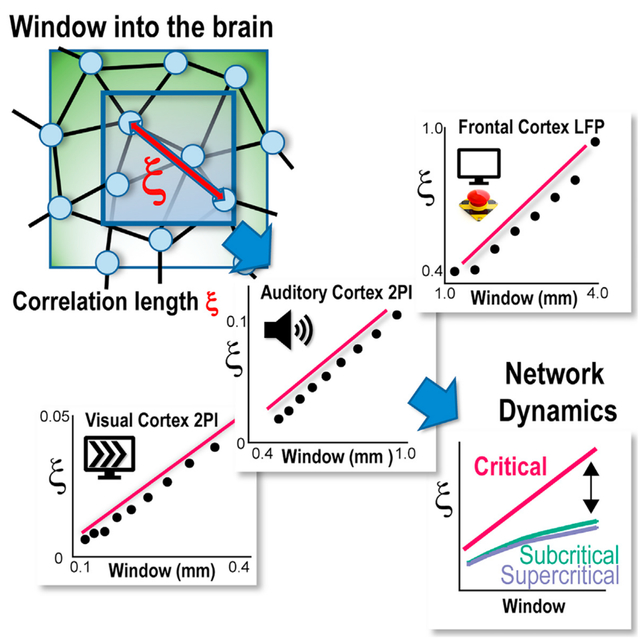

## INTRODUCTION

Even simple stimuli or movements engage large numbers of neurons in the mammalian cortex. These robust population responses are contrasted by the heterogeneity and fluctuations in responses of single neurons observed even during repeated sensory stimuli or motor outputs. This so-called trial-by-trial variability has been consistently found *in vivo* for neurons that are highly selective to a particular stimulus feature^1–4^ as well as for non-selective neurons.^5^ Response variability has been demonstrated *in vitro* under network and stimulus conditions of reduced complexity, supporting the notion that this variability is an essential feature of cortical networks (e.g., Haroush and Marom^6^). While part of the variability has been attributed to single-neuron properties,^7,8^ numerous findings point to a significant role of the network dynamics in the observed variability. Response variability correlates with ongoing neuronal activity preceding a stimulus,^9^ is shared among neurons,^10,11^ includes interactions between selective and non-selective neurons,^12^ is supported by local cortical motifs such as fan in,^13^ and depends on the balance of excitation and inhibition.^6^ Response variability has also been found to correlate with macroscopic aspects of the animal such as animal movement and behavioral performance.^14^ Given the many potential sources contributing to trial-by-trial variability, it is currently not clear whether there are overarching principles that robustly quantify the spatial organization of this response variability among neurons.

Correlation length measures have been successfully applied to identify the relationship between local, fluctuating system components and global, coherent system responses.^15^ This approach has been used to demonstrate that spontaneous fluctuations in the blood-oxygenated signal scale linearly with the size of the brain region measured.^16^ Going beyond neuroscience, a linear relationship between correlation distance and system size has been identified in the context of bird flocks, where a flock still maintains a coherent trajectory in space despite fluctuations in single bird trajectories.^17–19^ Using neuronal simulations, we recently demonstrated that correlation distance measures can be obtained by either exploring systems of different sizes or increasing the window of observation for a large network of fixed size.^20^ This latter approach suggests that correlation measures can be extended to *in vivo* studies of neuronal dynamics, in which brain size is fixed while the spatial window to study the neuronal population can be changed.

Here, we evaluated the correlation length for cortical trial-by-trial variability in the primary visual (V1) and auditory cortex of awake mice at the microscale using 2-photon imaging (2PI). We demonstrate that neuronal fluctuations within trials are spatially correlated and exhibit a linear scaling in correlation length with the size of the cortical region observed. We expand our findings to the mesoscale in the prefrontal (PF) and premotor (PM) cortex of non-human primates by measuring responses in the local field potential (LFP) to cued and self-initiated motor behavior using chronically implanted high-density microelectrode arrays (MEAs). Our results identify a robust form of spatial scaling in the trial-by-trial variability for primary and higher cortical areas *in vivo* in line with our simulations of neuronal responses in networks exhibiting critical dynamics. We suggest this spatial scaling in correlated variability to be in line with critical dynamics in the cortex, which optimizes information processing.

## RESULTS

### The correlation length in mouse V1

**Figure 1.**
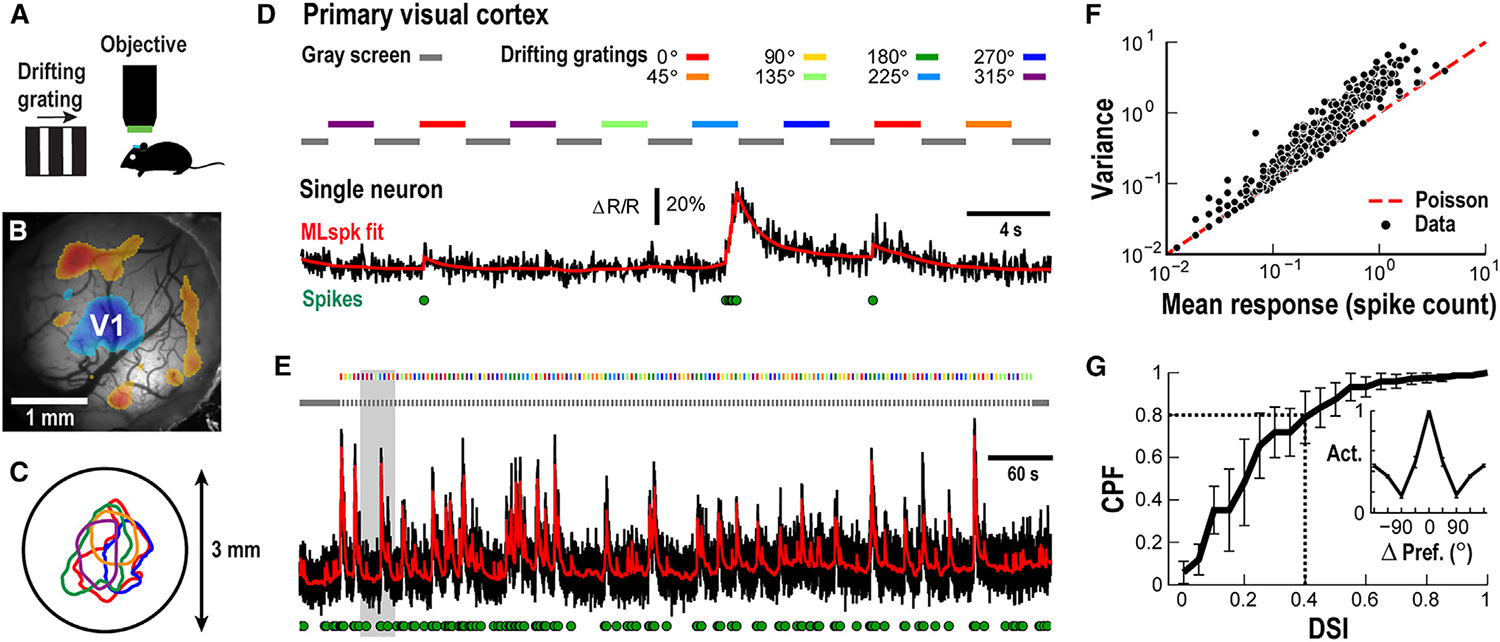
Trial-by-trial variability and tuning in single neurons during visually evoked activity in the primary visual cortex (V1) of an awake mouse (A) Sketch of stimulation and head-fixed 2PI recording from an awake mouse. (B) 3 mm craniotomy window with overlaid sign map identifying the V1 in blue (single mouse; see STAR Methods). (C) Identified V1 patch for 5 different mice (colors) and their position relative to the 3 mm craniotomy window (circle). (D) Variable response of a single V1 pyramidal neuron to semi-random presentation of 2 s large-field drifting gratings at 8 directions. Black: ΔR/R; red: MLspike fit of ΔR/R; green circles: estimated spikes (see STAR Methods). Color bars: stimulus direction. Gray bar: contrast-matched gray screen. (E) Full recording of single neuron to semi-random presentation of 8 stimuli. Gray area: enlarged period shown in (D). (F) Trial-by-trial variability of single neuron responses (dots; n = 228 cells across n = 7 animals), quantified by dividing response variance by response average, exceeds prediction from a Poisson process (red broken line). (G) About 20% of responding neurons show a direction-selective index (DSI) above 0.4. Cumulative probability function (CPF) for n = 7 animals (mean ± SD). Inset: neurons with a DSI >0.4 show minimal response at orthogonal directions. Mean direction selectivity profile normalized to corresponding preferred direction (n = 47 cells; n = 7 animals; mean ± SD).

For cellular analysis of the trial-by-trial variability, the spiking responses of pyramidal cells were recorded in the functionally identified V1^21^ of awake mice (Figures 1A–1C; n = 7 mice). Mice were quietly resting during recordings while 2-s-lasting drifting gratings were presented every 4 s for 8 different directions, separated by luminance-matched grayscale screens (Figures 1D and 1E). Using 2PI and the genetically encoded calcium indicator (GECI) Yellow Chameleon (YC2.6), we recorded from areas of L×L=~400×400μm in superficial cortical layers at depths of ~100–150 μm from the cortical surface, resulting in simultaneous recordings of up to ~150 neurons. High trial-by-trial variability was found in the response of many single neurons,^22^ as demonstrated by a variance larger than one relative to the average spike count (Figure 1F). This variability was present with ~20% of neurons being tuned to the stimulus direction with a selectivity index larger than 0.4 and exhibiting minimal responses orthogonal to their preferred orientation (Figure 1G; see STAR Methods).

**Figure 2.**
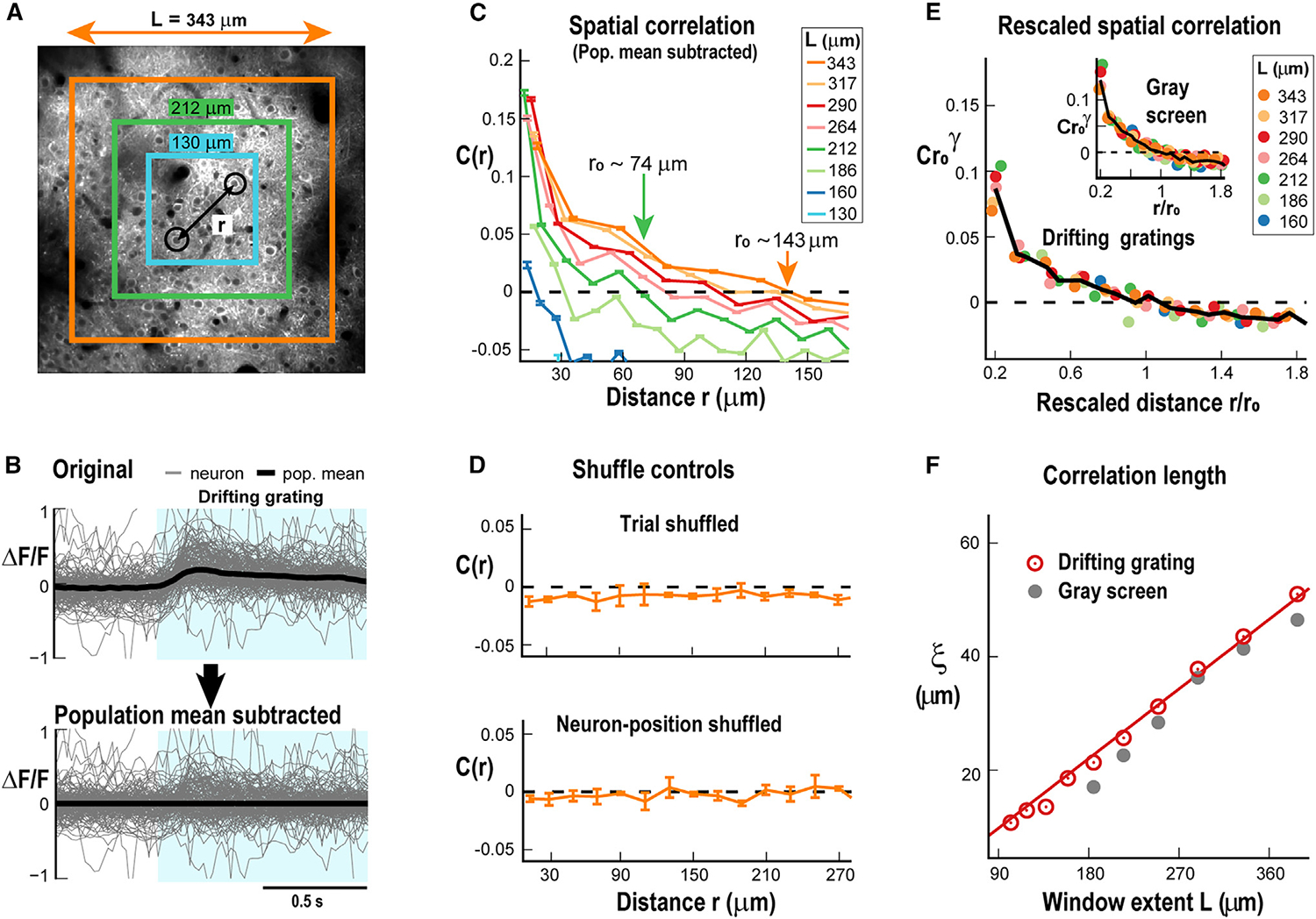
Neuronal fluctuations around the population response exhibit within-trial spatial correlations that grow with the window of observation (A) Example of a 400 × 400 μm 2PI showing Yellow Chameleon 2.6 (YC2.6) expressing pyramidal neurons in V1 (~120 μm cortical depth). Example to measure pairwise correlation between two V1 neurons (black circles) separated by distance r for 3 windows of different lengths *L*. (B) Top: overplot of single neuron responses (gray) and average neuronal response (black) for a single trial. Bottom: same overplot of single neuron responses with the average neuronal response for that trial subtracted. Blue shaded region denotes stimulus on. Note the remaining high fluctuations in single neuron responses after subtracting the spatial average (full window of recording). (C) Correlation in the fluctuations around the mean of activity during visual stimulation decays with distance and crosses zero. The zero crossing defines *r*_0_, which is seen to increase with window length (arrows). Mean ± SD for n = 7 mice. Colors: window length. Broken line: zero correlation. (D) Spatial correlations in fluctuations around the mean are specific to each trial and are abolished by trial shuffling (top), which is similar to shuffling neuronal positions (bottom). *L* = 343 μm case is shown for n = 7 mice (mean ± SD). (E) Scale-invariant spatial correlation functions in V1 during sensory stimulation obtained by collapsing correlation functions for *L* ≥ 160 μm for drifting gratings and gray screen (inset). Rescaled correlations ${Cr}_{0}^{\text{γ}}$ as a function of distance normalized by *r*_0_. Black solid line: average approximating the general function *F* in Equation 1. (F) Linear increase in correlation length ξ with window extent *L* in V1 in response to drifting gratings (red) or during gray screen (gray). Red solid line: linear regression for drifting grating (p = 2:4×10^−3^ chi-squared test; see STAR Methods).

We next studied what type of spatial correlations exist within this variability (Figure 2A) by examining, for each trial, the deviation of a neuron’s response from the population response (Figure 2B, top). Accordingly, we subtracted the spatial population average as a function of time from each neuron’s time series (Figure 2B, bottom) and calculated the mean pairwise correlation in the deviations across neurons as a function of distance r, C(r). In this approach, the population response depends on the size of our cortical observation window, an *ad hoc* experimental constraint in 2PI. Thus, we systematically calculated C(r) for different sizes of our squared observation window quantified by its length L. The resulting series of functions C(r,L) (Figure 2C) decayed with distance and eventually turned negative, i.e., neuronal responses became anticorrelated around the mean. These robust functions, which cross zero at increasing distances for windows of increasing size (r_0_; Figure 2C, arrows), show that the residual fluctuations of individual neurons around the population mean are significantly correlated over distances, as further demonstrated by their disappearance after trial shuffling or shuffling neuronal positions directly (Figure 2D). Furthermore, we found that C(r) decayed similarly for larger windows, and we achieved a full collapse of C(r,L) for L≥160μm (Figure 2E; n = 7 mice; see also STAR Methods) both for drifting gratings and gray screen conditions, i.e., spontaneous activity. This collapse can be expressed as^17^

Equation 1 $$C\left(\frac{r}{r_{0}(L)},L\right)\cdot {\left[r_{0}(L)\right]}^{\gamma }=F\left(\frac{r}{r_{0}(L)}\right),\;$$

where γ is calculated from the derivative of C at $r_{0}$ (see STAR Methods) and F is a dimensionless scaling function that does not depend on the window extent L (as approximated by the average in Figure 2E, black line).

### Linear scaling of correlation length in mouse V1

The correlation length, ξ, can then be obtained as^23^

Equation 2 $$\xi^{2}(L)=\frac{\int_{0}^{r_{0}(L)}\;\;r^{2}C(r,\;L)dr}{\int_{0}^{r_{0}(L)}\;\;C(r,\;L)dr}$$

(see also STAR Methods). How the estimated correlations between neurons change with spatial window size L, ξ(L), has been found in simulations to be informative about the underlying network dynamics.^20^ Specifically, linear scaling with the spatial extent of the observed system has been found for critical dynamics, in contrast to a sublinear scaling linked to sub- or supercritical dynamics.

**Figure 3.**
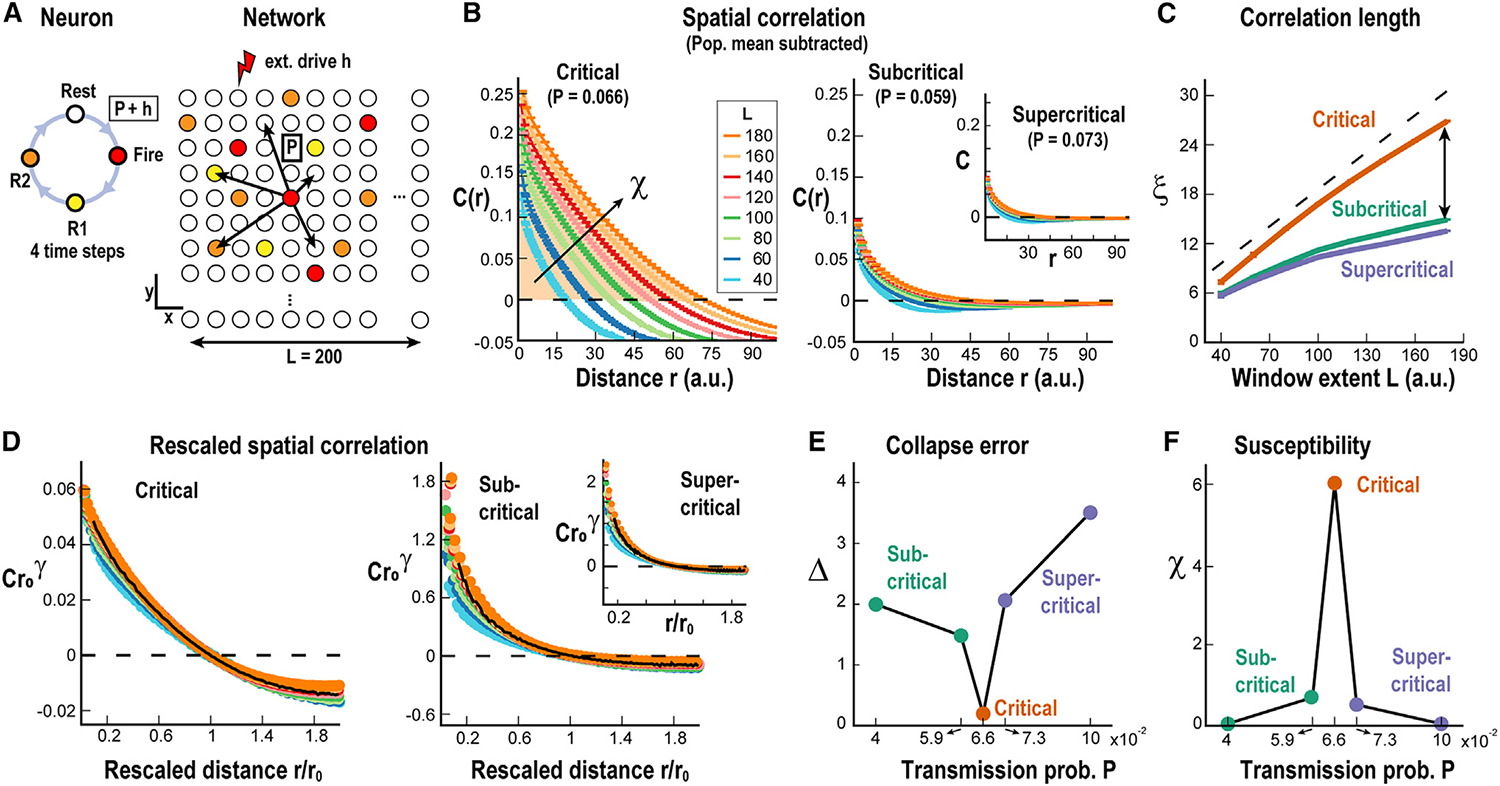
Neuronal model identifying linear scaling in correlation length to be unique to critical dynamics (A) The model consists of an *L* × *L* grid of units, whose state at each time step can be resting, firing, or refractory (Rest, Fire, R1, R2). A resting unit can fire with probability *h* = 10^−7^ or via a firing neighbor with probability P, which can be adjusted to make the network critical. Units are connected with a probability that decays with distance as *P*_conn_ = *e*^*r*/5^, where *r* is the distance between the units and the connectivity *K* is set to 8. (B) Correlations in activity fluctuations as a function of distance for critical (P = 0.066, left), subcritical (P = 0.059, right), and supercritical regime (P = 0.073, right inset). Color code: the linear size of the observed window or grid extent *L*. Broken line: zero correlation. Mean ± SD. (C) Correlation length ξ as a function of grid extent *L* for subcritical (green), critical (orange), and supercritical (purple) networks. Dashed line: linear growth as a visual guide. Note that *ξ* approaches linear scaling for the critical network while asymptoting outside criticality (green and purple). (D) Same as (B) but rescaling the axis by the correlation zero crossing *r*_0_. (E) Collapse error *Δ* is minimized at criticality (orange). (F) Susceptibility *χ*, calculated as the area under the correlation function *C(r)* up to *r*_0_ (see arrow and shaded area in B), has a sharp peak at criticality.

As can be seen in Figure 2F, ξ(L) scaled linearly with L both during gray screen as well as during drifting gratings (p = 2.4×10^−3^ chi-squared test for comparing the obtained linear regression to the data vs. the chi-squared distribution; see STAR Methods). We were able to capture the distance-dependent scaling of ξ(L) in a balanced neuronal network with local synaptic interactions and critical dynamics (Figure 3). In these simulations, neurons were connected locally and could induce firing in neighboring, non-refractory neurons with probability P. The network of 200 × 200 neurons was driven by a low external rate h, which induced a low probability of spontaneous firing (Figure 3A; see STAR Methods). Changing P allowed the network to be tuned to subcritical, critical, or supercritical dynamical regimes (Figure 3B). We found that ξ(L) grew close to linear in the critical regime, whereas growth was more sublinear, with asymptotic ξ in the sub- and supercritical regimes (Figure 3C). Furthermore, we showed that the collapse of C(r,L) was best in the critical regime (Figures 3D and 3E), at which the sensitivity of the network to external input, i.e., susceptibility, was maximized (Figure 3F). These results demonstrate robust scaling in long-range correlations despite high trial-by-trial variability in the V1 in line with predictions from locally interacting neurons establishing critical network dynamics.

### Scaling of correlation length in mouse auditory cortex

**Figure 4.**
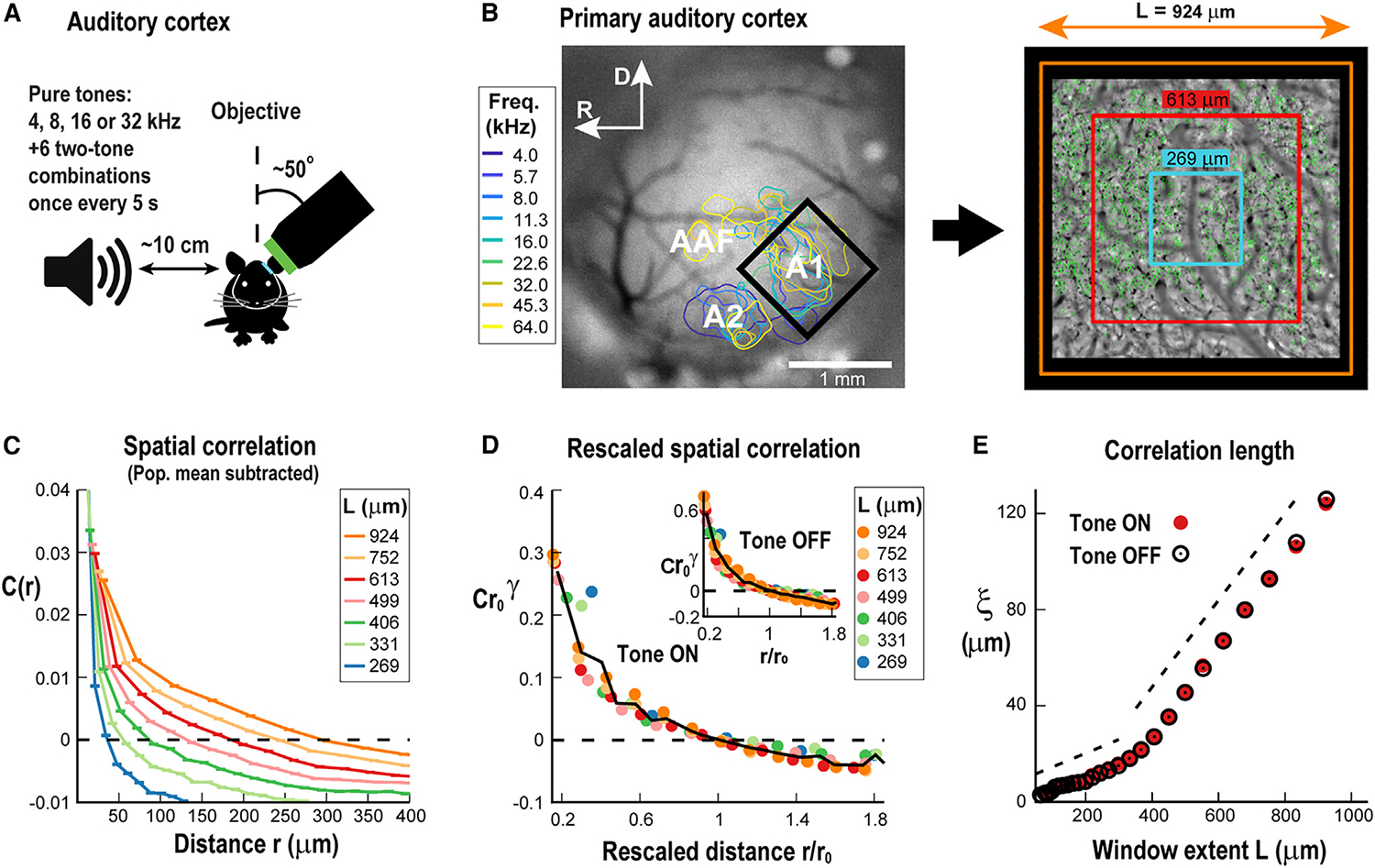
Linear scaling in correlation length for the auditory cortex (A) Sketch of auditory stimulation during 2PI recording of a head-fixed awake mouse. A pure tone or two-tone combination is presented every 5 s using a microphone 10 cm away from the mouse’s right ear while simultaneously recording from A1 in the contra-lateral hemisphere. (B) Left: example craniotomy window with tonotopic mapping of auditory areas (A1, primary auditory cortex; A2, secondary auditory cortex; AAF, anterior auditory field). A black rectangle outlines the borders of the 2PI view of the A1. Right: example ~1 × 1 mm area of 2PI aligned to the wide-field craniotomy window on the left with windows of different extent. Small green regions show labeled neurons. (C) Correlation in the fluctuations around the mean in response to auditory stimuli decays with distance and crosses zero, similarly as shown for V1 (mean ± SD for n = 11 mice). Colors: window length. Broken line: zero correlation. (D) Collapsed correlation functions for different *L* (color code) during tone ON (main) and tone OFF (inset) (averages for n = 11 animals). Broken line: zero correlation. (E) Correlation length scales linearly with window extent during tone ON (red) and tone OFF (black).

We extended our findings from the V1 to the auditory cortex in awake mice (Figure 4) using a window up to 4 times larger and close to ~1 × 1 mm (Figures 4A and 4B). Awake mice passively listened to short (1 s) auditory stimuli (pure tones or a combination of two tones between 4 and 32 kHz) presented every 5 s.^24,25^ Calcium responses were recorded in pyramidal neurons from superficial layers (n = 11 mice; ~900 cells on average per recording) using 2PI and the GECI GCaMP6s.^26^ We calculated the correlation functions C(r) from evoked responses for different recording window sizes, L, to obtain correlation lengths, ξ (Figures 4B and 4C). As demonstrated for the V1, the corresponding correlation functions C(r,L) in the primary auditory cortex (A1) could be collapsed for L≥269μm and were similar for tone-OFF and tone-ON conditions (Figure 4D). Importantly, the correlation length ξ was found to scale linearly with L during both tone-OFF and tone-ON conditions (Figure 4E; p = 0.025 chi-squared test for comparing the obtained linear regression to the data in the 400–1,000 μm range vs. the chi-squared distribution; n = 11 mice; see also STAR Methods).

Our findings demonstrate that spatial correlations in trial-by-trial fluctuations around the population mean between neurons in the visual and auditory cortex do not exhibit a finite length but, instead, scale linearly with the size of the observation windows.

### Scaling of correlation length in the frontal cortex of behaving non-human primates

**Figure 5.**
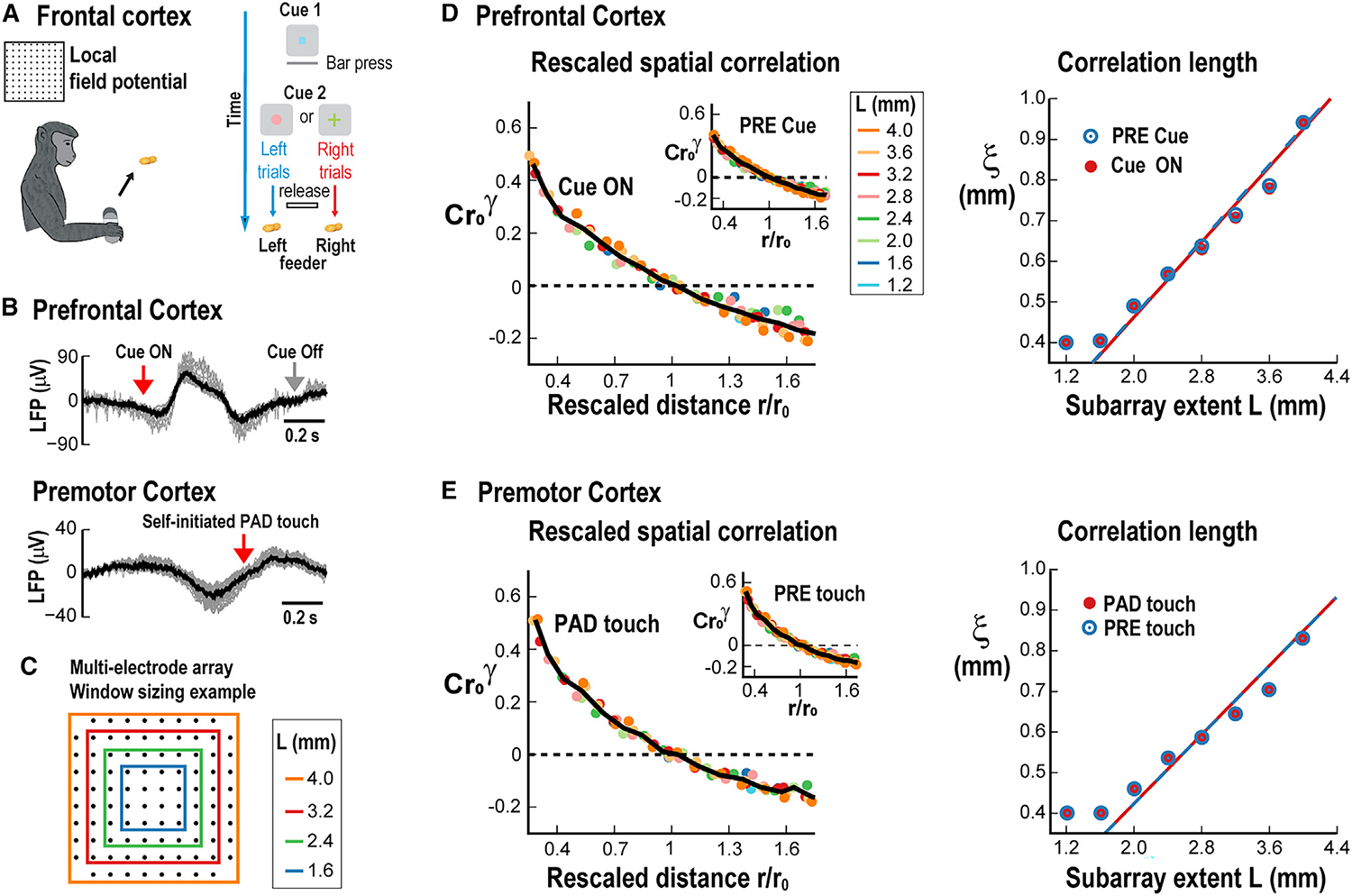
Linear scaling in correlation length for the frontal cortex in behaving non-human primates using multi-electrode array recordings of the local field potential (LFP) (A) Sketch of experimental design for monkey tasks. Left: monkey performs self-initiated pad touch for rewards while premotor cortex is recorded. Right: working memory task in which the monkey has to approach the correct feeder based on a cue while activity from the prefrontal cortex is recorded through a multi-electrode array. (B) Overplot of LFP traces from a single trial with single electrodes (gray) and population average over all electrodes (black). Top: prefrontal cortex after visual “cueon” in preparation for motor movement. Bottom: premotor cortex for self-initiated pad touch (arrow). (C) Example schematics to group-adjacent electrodes into square subarrays. (D) Left: collapsed correlation functions for the different subarray sizes (colors) during “cue on” and “pre” (inset) periods. Right: linear growth of *ξ* for “cue on” (red) and “pre” (blue) periods. Line: linear regression. (E) Same analysis as in (D) for self-initiated movement in premotor cortex. For all linear regressions in (D) and (E), chi-squared test p < 10^−4^.

To further evaluate the robustness of our findings in neuronal response variability, we studied trial-by-trial variability in cortical responses of behaving non-human primates. To expand scaling in correlation length to the cortical mesoscale, we employed high-density MEAs (10 × 10 electrodes without corners; 400 μm interelectrode distance) and recorded the LFP in PF (monkey A) and PM (monkey B) cortex (Figure 5A; for details, see Yu et al.^27^). Monkey A was trained in a visual-motor mapping task (Figure 5B, top; see STAR Methods), while monkey B performed a self-initiated movement task (Figure 5B, bottom). We applied the methods described in the previous sections to investigate whether similar findings could be established at this larger scale. Amplitude fluctuations in the LFP were obtained by subtracting the instantaneous average on the squared subarrays of width L from each electrode LFP (Figure 5C). We then obtained C(r,L) between pairs of electrodes for the subarray of width L in multiples of the interelectrode distance. C(r,L) decayed with distance (Figure S1) in remarkably similar shapes for different L, allowing for the successful collapse of the curves (Figures 5D and 5E, left). The collapsed functions were similar among both monkeys during baseline and task-evoked activity (Figures 5D and 5E, cp. insets). As found for the V1 and auditory cortex, ξ scaled linearly with L for both cortical regions and was similar between baseline and sensory/motor processing epochs (Figures 5D and 5E, right; p < 10^−4^ chi-squared test for all cases). Importantly, this was not the case when trial shuffling was employed, as that procedure leads to a non-decaying near-zero correlation function (Figure S1), in line with results obtained for cellular trial-by-trial shuffled responses in our 2PI experiments from mice.

### Linear growth of the correlation length can be misleading in the presence of common input

To highlight the importance of subtracting population averages when calculating the correlation length, we reanalyzed the data without that step. In this approach, we simply obtain the Pearson’s pairwise correlation for every pair of neurons/channels, from which we calculate the correlation function C(r) by averaging correlation values from the same distance between neurons/channels. The observed function decays as a power law with distance (Figure S2), which is in line with expectations from criticality theory,^28^ but the function is noisy and subjected to finite-size effects. When studying the Pearson’s pairwise correlations as a function of window size (Figure S3), we note that the curves for different L follow the same general function, with the difference being a trivial limitation of the maximum distance between neurons that can be observed for different window extents (Figure S3A, top). When calculating ξ for the V1 dataset (Figure S3A, bottom), we cannot see the expected linear growth with L. When trial shuffling the data (Figure S3B, top), despite C(r) being close to zero for most distances, ξ still follows a similar function of window extent (Figure S3B, bottom). Similarly, when studying our non-human primate PF cortex datasets (Figure S4), we find that C(r) remains above zero even after trial shuffling (cp. Figures S4A and S4B, top). These residual correlations must be related to common input to those channels since the only signal maintained after trial shuffling is the average input from the cue presentation. In this case, the non-zero residual correlations lead to a linearly scaling ξ(L), regardless of trial-shuffling (Figures S4A and S4B, bottom; p < 10^−4^ chi-squared test for both cases). We note that when employing the original method to calculate the correlation functions, that is, removing the population average from each channel, trial shuffling resulted in near-zero correlations across all distances (see Figures 2D and S1). In the brain, where external drive is constantly present, our results highlight the importance of reducing the impact of common input when estimating inter-neuronal correlations. Our findings demonstrate that this can be achieved by a simple subtraction of the population average as a function of time.

## DISCUSSION

Trial-by-trial variability transcends all sensory and motor responses in systems neuroscience and has been successfully linked to many domains, from single neurons to networks to behavior. Our comparative study assessed trial-by-trial variability for different sensory dimensions (2-dimensional visual space vs. 1-dimensional auditory space), spatiotemporal scales (microscale in 2PI vs. mesoscale in array recordings), cortical areas (primary vs. frontal cortex), sensory vs. motor responses, experimental conditions (2PI vs. LFP), and species (mouse vs. non-human primate). Our identification of a common scaling function that is sensitive to trial shuffling suggests an overarching principle in the correlations that govern trial-by-trial variability.

### Linear growth in correlation length indicates systemwide correlations among neurons

If the distance over which neuronal activities correlate were to be finite, e.g., <100 μm, then our approach would have revealed an upper bound in window size, or cortical area, for which the correlation length saturated. Instead, for all our experimental approaches—first, for up to 400 μm at the level of individual neuronal firing in the V1, then extending for up to 1 mm in the auditory cortex using 2PI, and, finally, for up to 4 mm in the LFP in awake non-human primates using high-density arrays—we demonstrated that the correlation length scales linearly with the extent of the cortical area observed. These results identify a dynamical framework that captures the intracortical spatial correlations that coexist with high trial-by-trial variability in layer 2/3 of the cortex. Such scale-free correlations signify that these layers exhibit inter-neuronal correlations that can span the whole network over long distances, i.e., neuronal activity from local interactions significantly correlates with and thus might influence activity at far distant cortical regions, even if these regions are not directly connected through long-range connections. Such an organization in spatial correlations could be of an advantage for information processing when local information from different cortical sites needs to be integrated for higher-order cortical functions. As outlined further below, our results strongly suggest that critical dynamics in layer 2/3 of the cortex give rise to the observed scaling in spatial correlations.

### Removal of the spatial average uncovers inter-neuronal correlations unique to single trials

We used the fluctuations of local neuronal activity around the instantaneous mean of the observed network to evaluate its correlation length from individual trials. The subtraction of the (observed) population average before calculating correlations has been successfully applied in, e.g., the context of bird flocks,^17^ where one needs to evaluate how birds move about one another, disregarding the overall movement of the flock. This approach is more common in physics and, to our knowledge, has been applied here for the first time to single neurons and local neuronal populations in awake animals to study evoked responses. While removing the average spatial velocity is readily interpreted in the context of a moving flock, the impact of removing the spatial average is more difficult to assess with respect to brain activity. Our trial-shuffling controls provide an important step in that direction. In these controls, the pairwise correlation between neurons i and j is calculated from responses to different trials, which removes any within-trial correlations between them, while common correlations are maintained. We clearly demonstrated that (1) trial shuffling destroys any spatial dependencies equivalent to randomizing the position of neurons themselves and (2) if the spatial average is not subtracted, then the resulting correlation functions demonstrate similar growth in the original presentation or after trial shuffling. Our results show that subtracting the spatial average focuses our analysis on identifying the organization of local cortical correlations that cannot be explained from common input. Importantly, in contrast to previous work on ongoing activity,^16^ our work clearly links the spatial and linear growth of connected correlations to that of sensory- and movement-evoked neuronal activities, i.e., during times of information processing. To ensure the reliability of the method used to calculate the correlation length, we compared our results, which were obtained using a more traditional method, to those previously obtained in the literature.^16,17^ In contrast to the method employed so far, which identifies the zero crossing of the correlation function, here, we are using an integral approach, restricting the integration to positive C(r) values $\left(C(r_{0})\;=\;0\right)$. Note that, analytically, assuming the family of C(r,L) curves can be collapsed into a single function that does not depend on L (see Figures 2E, 4D, 5D, and 5E), it can be shown that the correlation lengths obtained by the two methods are proportional (see STAR Methods).^16,17^ However, the required collapse in scaling functions cannot be *a priori* assumed for the general case.

### The correlation length identifies robust spatial dependencies in neuronal correlations that differ from common input

“Noise” correlations have been commonly identified as inter-neuronal correlations that are not due to common input.^29,30^ “Noise” correlations are typically calculated between a small number of neuronal pairs, making it difficult to identify spatial dependencies that arise from inter-neuronal interactions. In contrast, our present analysis readily obtains robust spatial dependencies in correlations that cannot be explained by common input. Importantly, these spatial dependencies can be readily collapsed; thus, the resulting functional is not dependent on the windowing procedure itself. The collapse was found for all studied datasets. These results corroborate what was found in simulations of critical networks^20^ when comparing correlation functions obtained from the complete system to those obtained from a window into those systems, where the obtained collapse implies that correlations missed due to neurons being outside the window of observation do not add up to a change in the underlying correlation function, i.e., within-window and outside-window correlations follow the same spatial structure. We conclude that our analysis provides a robust estimate of spatial functions that describe inter-neuronal correlations. The exact relationship between traditional “noise” correlations and connected correlations reported here are beyond the scope of the current study.

### Linear growth in correlation length in superficial layers of the cortex

Our findings of a similar growth in connected correlations when using spikes (2PI recordings) or the LFP (MEAs) might be specifically attributable to cortical dynamics in superficial layers, for which several studies demonstrated a relatively tight correspondence between the local firing rate and the LFP, the latter reflecting largely local, subthreshold activity. Rasch et al.^31^ successfully reconstructed extracellular spike trains based on the ongoing LFP in superficial layers of the V1 of non-human primates. Petermann et al.^32^ showed that the negative LFP amplitude monotonically increases with the local firing rate and the synchronization of extracellular units in superficial layers of the PM and PF cortex in awake non-human primates. At the cellular level, neuronal avalanche dynamics in layer 2/3 of mouse and rat, whether recorded with GECIs reflecting suprathreshold spiking^33^ or with genetically encoded voltage-sensitive dyes reflecting largely subthreshold activity,^34^ reveal similar statistical changes to anesthesia. These studies are in line with our findings reported here that, in the superficial cortex, both the LFP and spiking in local neuronal groups demonstrate a similar growth in connected correlation yet demonstratively at largely different spatial scales.

That the cortical state is known to affect trial-by-trial variability^35^ and correlation among neurons^10,36^ has been a longstanding observation, and part of this variability originates from the cortical network itself.^11,37–39^ It is well established that the correlation length diverges at criticality in the thermodynamic limit,^15^ which, for systems of finite size, can be demonstrated by showing that the correlation length grows with system size. Our demonstration of linear scaling in correlation length is in line with findings in network simulations with critical dynamics^20^ and our simulations in the present study.

### Trial-by-trial variability is in line with the cortex operating in a critical regime to improve information processing

Critical dynamics has been a fundamental driver in understanding the optimization of information processing in complex systems considering the evidence that fluctuations or variability are high at criticality (e.g., Fraiman and Chialvo,^16^ Shew et al.,^40^ and Tkacik et al. ^41^). Decades ago, it was suggested that critical dynamics optimize information transfer in gene-regulation networks.^42^ For the brain, highly desirable aspects of information processing have been shown to improve at criticality, such as the maximization of mutual information between stimulus input and output,^43–47^ increased information capacity (i.e., the number of possible internal states a network can establish),^40,48,49^ improved stimulus discrimination,^50,51^ and the ability of neurons to flexibly change synchronization while maintaining an overall robust degree of phase locking.^52–55^

In conclusion, our findings support the notion that trial-by-trial variability, rather than reflecting pure noise, might represent an intrinsic property of critical cortical networks during information processing.

### Limitations of the study

We evaluated the correlation length as a function of subsamples of the recorded region, i.e., compact windows, and not as a function of system size, which is the more common approach in physics. While it is known that windowing differs to a certain degree from finite-size effects,^56^ it has been shown recently for two different models that such a “box scaling” Ansatz^20^ leads to similar results for networks with critical dynamics. However, it is important to note that differences in the scaling of the correlation length for systems slightly outside criticality can be hard to distinguish,^20^ and thus one needs caution when interpreting the observed scaling in regard to originating from a critical system.

## STAR★METHODS

### RESOURCE AVAILABILITY

#### Lead contact

Further information and requests for resources and reagents should be directed to and will be fulfilled by the lead contact, Dietmar Plenz (plenzd@mail.nih.gov).

#### Materials availability

This study did not generate new unique reagents.

#### Data and code availability

- The data reported in this paper will be shared by the lead contact upon request.
- All original code has been deposited at GitHub and is publicly available as of the date of publication. DOIs are listed in the key resources table.
- Any additional information required to reanalyze the data reported in this work paper is available from the lead contact upon request.

### EXPERIMENTAL MODEL AND STUDY PARTICIPANT DETAILS

#### Animals

All procedures followed the Institute of Laboratory Animal Research (part of the National Research Council of the National Academy of Sciences) guidelines and were approved by the NIMH Animal Care and Use Committee or by the Johns Hopkins University Institutional Animal Care and Use Committee. Wild type (C57BL/6J) mice or F1 offspring of CBAxThy1-GCaMP6 (adult, males and females) housed under a reversed 12 h-light/12 h-dark cycle with *ad libitum* access to food and water were used in the two-photon imaging part of the study. Adult rhesus monkeys (*Macaca Mulatta*, a 9 years-old male and an 8 years-old female) were used in the electrophysiology part of the study.

### METHOD DETAILS

#### Mouse surgery and preparation

Wild type (C57/Bl6, Jackson Laboratory) mice (V1) or F1 offspring of CBA (Jax# 000654)xThy1-GCaMP6 (Jax#024275; A1),^26^ were housed under a reversed 12 h-light/12 h-dark cycle with *ad libitum* access to food and water. Imaging experiments were generally performed near the end of the light and the beginning of the dark cycle. A custom-made titanium head bar was surgically implanted onto the skull of the mice under isoflurane anesthesia (4% induction, 1–1.5% maintenance). A circular craniotomy (~3 mm) was made above the area of interest (visual or auditory cortex). For V1 experiments, a virus containing the genetically encoded calcium indicator YC2.6 was injected at a depth of ~250–300 μm. After that, a cranial window composed of two 3 mm diameter coverslips glued to a 5 mm coverslip was implanted and the entire area (except for the window) was sealed with dental cement.

#### Identification of V1 maps

Retinotopic maps of V1 were generated for individual mice before recording using published methods.^21^ Briefly, awake, head-fixed mice faced with their left eye a 19″ LCD monitor placed at 10 cm distance and tilted 30 toward the mouse’s midline. Using Psychophysics toolbox,^58^ contrast-reversing, spherically corrected checkerboard bars were drifted across the screen vertically (altitude) and horizontally (azimuth) for each of the four directions (30 repeats per direction). Simultaneous wide-field imaging (Quantalux, Thorlabs) captured YC2.6 fluorescence, which was averaged for each direction. Altitude and azimuth phase maps were calculated by phase-wrapping the first harmonics of the 1D Fourier transform for each of the four averages and subsequently subtracting the maps of the opposite directions. Sign maps were generated by taking the sine of the angle between the gradients in the altitude and azimuth maps as previously described.^21^ Borders were drawn around visual area patches and overlaid onto anatomical reference images to identify V1.

#### Visual stimulation and response measures

Visual stimuli were prepared in MATLAB using the Psychophysics Toolbox and delivered via a monitor (Dell, 60 Hz refresh rate) placed ~25 cm in front of the contra-lateral eye of the mouse. The stimulus was composed of moving gratings at 8 different directions presented for 2 s at maximum contrast, 0.04 cycles per degree and 2 cycles/s. Stimuli were interspaced by gray screen (matched for average luminance) for 2 s. Each direction was presented 20 times in randomized order, for a total of 160 iterations. We calculated the direction selectivity index using the common definition: $DSI=\left(R_{P}-R_{O}\right)/\left(R_{P}+R_{O}\right)$, where $R_{p}$ and $R_{O}$ are the responses to the preferred and opposite direction, respectively. Significance of DSI for each cell was assessed by comparing the values obtained from the original data with those obtained from shuffling the inter-spike intervals.

#### Acoustic stimulation

Sound stimuli were synthesized in MATLAB using custom software, passed through a multifunction processor (RX6, TDT), attenuated (PA5 Programmable Attenuator, TDT), and delivered via an ES1 speaker placed ~10 cm from the animal’s right ear (contralateral to the left brain hemisphere where imaging took place). The stimuli were generated as either pure tones, consisting of a single tone of 4, 8, 16 or 32 kHz, or six two-tone combinations of these (ten different stimulus types in total). 50 trials were repeated for each stimulus, resulting in 500 trials total presented throughout ~42 min with a 5-s inter-trial interval. SPL of all stimuli was calibrated to 70 dB (±3 dB) SPL using Brüel and Kjær Type 4944-A microphone and Type 1704 Signal Conditioner. ^24–26^

#### Two-photon imaging and analysis of the visual cortex

Images were acquired by a scanning microscope (Bergamo II series, B248, Thorlabs) coupled to a pulsed femtosecond Ti:Sapphire 2-photon laser with dispersion compensation (Chameleon Vision S, Coherent). The microscope was controlled by ThorImageLS software. The wavelength was tuned to 830 nm to excite YC2.6. Signals were collected through a 16× 0.8 NA microscope objective (Nikon). Emitted photons were directed through 535/22 nm (yellow) and 479/40 nm (cyan) band filters onto GaAsP photomultiplier tubes. The field of view was ~400 × 400 μm. Imaging frames of 512 × 512 pixels were acquired at 30 Hz by bidirectional scanning of an 8 kHz resonant scanner. Beam turnarounds at the edges of the image were blanked with a Pockels cell. The average power for imaging was <70 mW, measured at the sample. The obtained images were corrected for motion using dft registration software with MATLAB. Regions of interest (ROIs) were identified from the average image of the motion corrected sequence using custom code. For each labeled neuron, raw fluorescence signals over time were extracted from the ROI overlying the soma. The mean ratiometric signal, R, in each ROI was calculated across frames and converted to a relative fluorescence measure, $\Delta R/R_{0}$. The baseline signal R_0_ was estimated by using a sliding window that calculated the average fluorescence of points less than the 10^th^-percentile during the previous 1.3-s window (40 frames).

#### Two-photon imaging and analysis of the auditory cortex

The animal was placed in a holder under the microscope (Ultima 2Pplus, Bruker). We imaged a region previously identified as A1 by widefield imaging session of the whole cranial window.^24–26^ Two-photon imaging was done using a 16× 0.8 NA microscope objective (Nikon) and at an optical zoom of 1×. The field of view was of size 1109.9 × 1109.9 μm, yielding on average ~900 cells and acquired images had 1024 × 1024 pixels. The frame rate was 15 Hz. During the experiment, the head of the mouse was upright while the microscope nosepiece was rotated from the vertical position by 50–60° to match the angle of the cranial window surface. The imaging laser (Chameleon Discovery NX, Coherent) was tuned to a 920 nm wavelength to excite GCaMP6s. For analysis, we used the Suite2P package to perform motion correction, automated ROI detection, and raw cellular and neuropil fluorescence trace extraction.^57^ Fluorescent traces for individual ROIs from Suite2p were further analyzed with custom MATLAB scripts.

#### Monkey behavioral training and electrophysiological setup

Experiments were described previously.^27^ In short, two adult rhesus monkeys (*Macaca mulatta*) were surgically implanted with a titanium head post. After recovery, they were trained to sit head-fixed in a primate chair for behavioral performance. In the cue-initiated task, monkey A (male, 9 years old, 8 kg) had to press a bar in front of the chair upon presentation of the ‘trial-initiation’ cue on a computer screen. After ~2 s, the initiation cue was followed by an ‘instruction’ cue, for the duration of 1 s. Upon cue disappearance, monkey A had to release the bar and reach with his right arm to one of two specialized feeders, depending on which of two possible cues were presented. Approaching the incorrect feeder rapidly triggered a proximity sensor to sequester the food rewards in both feeders, which prevented the monkey from obtaining a reward on that trial. The inter trial interval was 3–5 s. In the self-initiated motor task, monkey B (female, 8 years old, 7 kg) had to move her right arm to touch a pad placed ~30 cm in front of the monkey chair after which a food reward was given. After the monkeys learned their respective tasks, a multi-electrode array (MEA; 96 channels, 10 × 10 without corners, inter-electrode distance 400 μm; electrode length 1 mm for monkey A and 0.55 mm for monkey B; BlackRock Microsystems) was chronically implanted in the arm representative region of the left prefrontal area (area 46, monkey A) or the left premotor cortex (monkey B). The LFP (1–100 Hz band-pass filtered; 2 kHz sampling frequency) was obtained from the implanted MEA. Electrophysiological signals as well as the timing of behaviorally relevant events, e.g., touching the pad, presentation of visual cues, etc., were stored for offline analysis.

#### Correlation analysis

The correlation of the fluctuations as function of distance^17^ was

(Equation 3) $$C(r)=\frac{1}{C_{0}}\frac{\sum_{i,j}\;\;u_{i}u_{j}\delta \left(r\;-\;r_{ij}\right)}{\sum_{i,j}\;\;\delta \left(r\;-\;r_{ij}\right)}$$

where $\delta \left(r-r_{ij}\right)$ is a smoothed Dirac δ function defining all pairs of neurons located at mutual distance r, $r_{ij}$ is the Euclidean distance from the i-th neuron’s spatial location to the spatial location of neuron j, and $u_{i}$ is the value of the signal v of neuron i at time t, after subtracting the overall mean of signals v from neurons inside the observation window of size L at that time $t:u_{i}(t)=\nu_{i}(t)-\overset{‾}{\nu }(t)$. To ensure that C(r=0)=1, the normalization factor $1/C_{0}$ was used. We note that since the instantaneous average is subtracted, C(r) is not equivalent to the commonly used pairwise Pearson correlation function.

The objective of computing the C(r) is to determine how the correlation length ξ changes with system size. Since system size, i.e., cortex area in our experimental data, was fixed, we investigated how ξ changes with system size subsampled by our recordings and neurons/electrodes within a window of length L. This proxy, known as “box-scaling” was validated recently by Martin and colleagues^20^ using neuronal network simulations and a ferromagnetic 2D Ising model. More specifically, for the 2PI data in mice, fields of view ranging from ~40 × 40 μm (windows with fewer than 5 units were ignored to avoid bias introduced by the average subtraction procedure when the number of units is too small) to the maximum possible size were considered, while for the monkey LFP data the smallest subarray considered was 3 × 3. To reduce noise effects, results were averaged across all possible subregions for any given size. The time series were smoothed in the time domain (using MATLAB routine *medfilt1.m* with 20 samples for the mice 2PI data, 8 samples for the monkey LFP data). This smoothing procedure improved statistics without changing the results qualitatively. To estimate the zero-crossing point more precisely for the experimental data, we fit 3^rd^ order polynomial functions to the C(r) curves around the zero-crossing.

To define the correlation length, we employed a traditional^23^ integral approach: $\xi^{2}\sim \int_{0}^{r_{0}}\;r^{2}C(r)dr/\int_{0}^{r_{0}}\;C(r)dr$, where $r_{0}$ is the zero-crossing of the correlation (see also Equation 2). We set C(0)=0, so effectively the integral starts at the shortest distance between units in the evaluated dataset. To quantify the linear growth in correlation length ξ as a function of window length L, we first obtained a linear regression of the ξ(L) data followed by chi-square statistics $\chi_{c}^{2}=\sum_{i}\;{\left[\xi \left(L_{i}\right)-R\left(L_{i}\right)\right]}^{2}/R\left(L_{i}\right)$, where $L_{i}$ is the i^th^ measured value of L and $R\left(L_{i}\right)$ is the linear regression value at $L_{i}$. $\chi_{c}^{2}$ can be used to obtain a p value that estimates how likely the data fit the linear regression that well by chance from the chi-square distribution. We rescaled the correlation vs. distance curves by normalizing the distances by $r_{0}$, the zero-crossing of the correlation function, and by rescaling the correlations by $r_{0}$ to the power of γ, calculated from the equation $\frac{dC}{dr^{\prime }}\left(r^{\prime }=1\right)\sim -L^{-\gamma }$ by fitting a power law to the derivative of C in respect to $r^{\prime }=r/r_{0}$ at $r^{\prime }=1$ as function of L to then estimate the slope γ.^17^ To obtain the collapse error Δ, we calculate the mean absolute difference between C(r,L) and the average curve after collapse across $L{\left\langle C(r,L)\times r_{0}^{\gamma }\right\rangle }_{L}$. Note that this renormalization does not have free parameters since all variables are obtained directly from the data, and therefore there is no minimization procedure for Δ as a function of γ. We note that, γ indicates how quickly correlations drop as function of distance and we have found it to generally be small (γ=0.13,0.44,0.87and0.74, on average, for the V1, A1, monkeys and critical simulation datasets). In the case of starling flocks,^17 γ^ was found to be close to 0.

#### Equivalance of correlation lengths and correlation distance with existing scaling collapse

Assuming correlation functions obtained at different windows L collapse as shown in the Results section, from Equation 1 we have:

$$C\left(\frac{r}{r_{0}(L)},L\right)=F\left(\frac{r}{r_{0}(L)}\right)\cdot {\left[r_{0}(L)\right]}^{-\gamma },$$

Then, with a variable change to $r^{\prime }=r/r_{0}$ and applying the integral formula for the correlation length in Equation 2, we have:

$$\xi (L)=\sqrt{\frac{\int C(r,\;L)r^{2}dr}{\int C(r,\;L)dr}}=\sqrt{\frac{\int F\left(r^{\prime }\right){\left[r_{0}(L)\right]}^{-\gamma }{\left[r^{\prime }r_{0}(L)\right]}^{2}r_{0}dr^{\prime }}{\int F\left(r^{\prime }\right){\left[r_{0}(L)\right]}^{-\gamma }r_{0}dr^{\prime }}}$$

$$\xi (L)=\sqrt{{\left[r_{0}(L)\right]}^{2}\frac{\int F\left(r^{\prime }\right)r^{\prime 2}dr^{\prime }}{\int F\left(r^{\prime }\right)dr^{\prime }}}=r_{0}(L)\sqrt{\frac{\int F\left(r^{\prime }\right)r^{\prime 2}dr^{\prime }}{\int F\left(r^{\prime }\right)dr^{\prime }}}$$

Therefore, $\xi (L)\sim r_{0}(L)$

#### Trial shuffling and spatial shuffling

Trial shuffling for the V1 data was obtained by randomly permuting the responses from each of the 8 presented directions separately. This was done for each neuron independently. Therefore, in each trial of the trial shuffled dataset activity from each cell corresponds to a response to the same stimulus presented in the original data but taken from different presentations of that stimulus.

#### Numerical simulations

We simulated a neural network, as described previously.^43^ In short, each neuron can be in one of three states at each time step: 0 for resting, 1 for active, and 2 and 3 for refractory. The model considers S^2^ neurons on a square lattice. Each neuron outputs to K other neurons, selected with an exponentially decaying probability function of the Euclidian distance r between them ($P_{Conn}\sim e^{-r/R_{0}}$, with $R_{0}=5$). A spatial cutoff is set in the interaction distance: neurons cannot directly connect at distances greater than $I_{c}=4R_{0}$ spatial units, the interaction length. Furthermore, to reduce small S effects, we employed periodic boundary conditions. Results were computed on a square grid of length L<<S, to reduce finite-size artifacts and to better mimic experimental data. A small Poisson drive ($h=10^{-7}$ per time step) to each neuron determined the overall rate of firing. The control parameter of the model determines the branching of the neural activity and was defined as σ=K×P, where P is the probability that an active neuron (i.e., in state 1) can excite each one of the K neighbors that it connects to. Therefore, as shown previously, the model was made critical by selecting a transmission probability P such that σ~1, for any given K (K=8 was employed for all simulations).

### QUANTIFICATION AND STATISTICAL ANALYSIS

Statistical analyses were performed using MATLAB. To evaluate the correlation length linear growth, the chi-square statistics was computed and used to obtain a p value from the chi-square distribution (see the Correlation analysis subsection above for more details). Relevant statistical values (number of animals *n* and p values) are reported in the corresponding figure legends as well as in the main text. Values are reported as mean ± standard deviation.

## Supplementary Material

1

## Figures and Tables

**KEY RESOURCES TABLE:** 

REAGENT or RESOURCE	SOURCE	IDENTIFIER

Bacterial and virus strains		

Yellow Chameleon (YC2.6)	NIMH, Bellay et al., 2015^33^	N/A

Experimental models: Organisms/strains		

Mouse: C57BL/6J	Jackson Laboratories	RRID:MGI:3028467
Mouse: CBA	Jackson Laboratories	RRID:IMSR_JAX:000654
Mouse: Thy1-GCaMP6	Jackson Laboratories	RRID:IMSR_JAX:024275
Monkey: Rhesus (Macaca Mulatta)	NIMH colony Poolesville	N/A

Software and algorithms		

MATLAB (various versions)	Mathworks	N/A
Suite2P	Pachitariu et al. 2017^57^	https://github.com/MouseLand/suite2p
Psychophysics Toolbox	Kleiner et al. 2007^58^	https://github.com/Psychtoolbox-3
Code for analysis	This paper	https://doi.org/10.5281/zenodo.10525350

## Highlights

- The cortex shows varying responses to repeated stimuli, termed trial-by-trial variability
- This variability reveals unique spatial correlations among neurons that differ from noise
- The corresponding correlation length grows linearly with the observed cortical area
- Such linear growth suggests cortex operates at criticality, improving information processing
