# Supplementary material for: Trial-by-trial variability in cortical responses exhibits scaling of spatial correlations predicted from critical dynamics
Source: Cell Rep. Author manuscript; Available in PMC 2024 Mar 21. (PMC10956720; doi:10.1016/j.celrep.2024.113762)
Supplement: 1 [file NIHMS1970894-supplement-1.pdf]

Cell Reports, Volume *43*

Supplemental information

Trial-by-trial variability in cortical responses exhibits scaling of spatial correlations predicted from critical dynamics

Tiago L. Ribeiro, Peter Jendrichovsky, Shan Yu, Daniel A. Martin, Patrick O. Kanold, Dante R. Chialvo, and Dietmar Plenz

Supplemental Information Inventory

Figure S1 (Related to Figure 5) Correlation functions in frontal cortex of nonhuman primates during movement execution and visual cue presentations and trial-shuffle controls.

Figure S2 Pearson's pairwise correlations decay with distance approximately as a power law.

Figure S3 Linear growth of the correlation length is insensitive to trial-by-trial interactions without population average subtraction for the V1 dataset.

Figure S4 Linear growth of the correlation length is insensitive to trial-by-trial interactions when common input is not removed for the prefrontal dataset.

Supplemental Information
Figure S1 (Related to Figure 5)

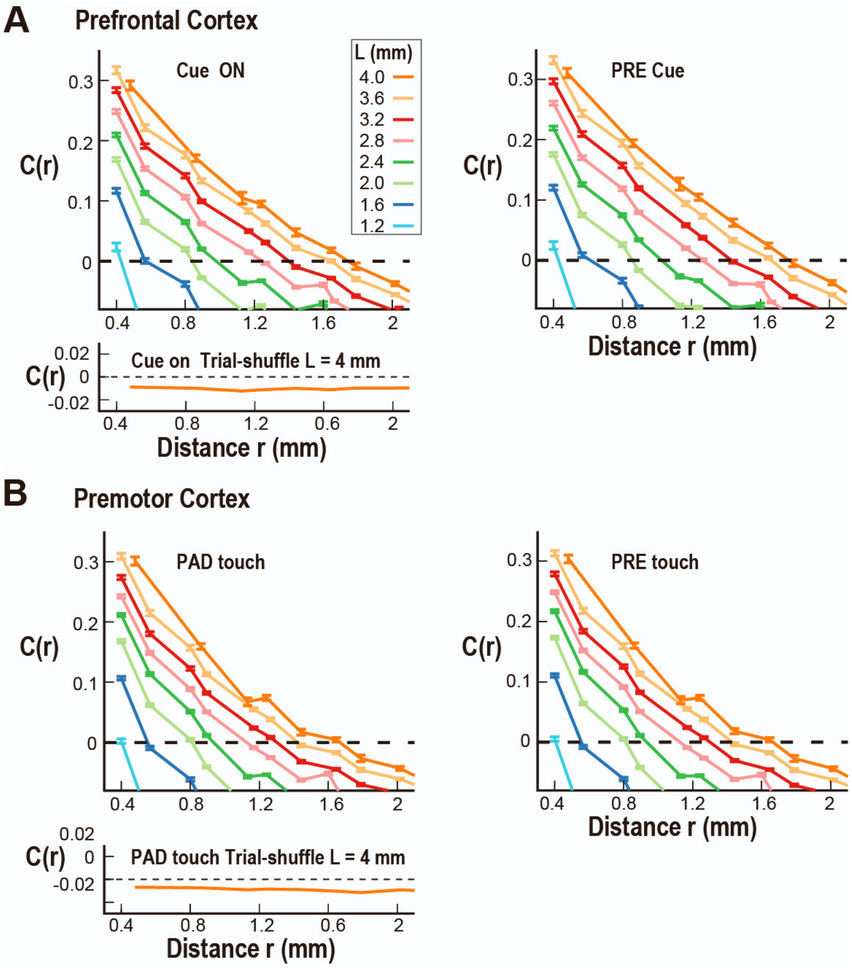

Figure S1 (Related to Figure 5) Correlation functions in frontal cortex of nonhuman primates during movement execution and visual cue presentations and trial-shuffle controls

(A) Non-collapsed average correlation in fluctuations around the instantaneous population mean as function of distance for different subarrays of side length *L* (*color code*). Prefrontal cortex (monkey B). *Left/right*: Pre/Cue on. *Black dashed line*: zero correlation. *Bottom right*: Near-zero correlations for trial-shuffled evoked datasets (*L* = 4 mm). (B) Non-collapsed average correlation in fluctuations around the instantaneous population mean as function of distance for different subarrays of side length *L* (*color code*). Premotor cortex (monkey A). *Left/right*: Pre/Pad touch. *Black dashed line*: zero correlation. *Bottom right*: Near-zero correlations for trial-shuffled evoked datasets (*L* = 4 mm).

Supplemental Information
Figure S2

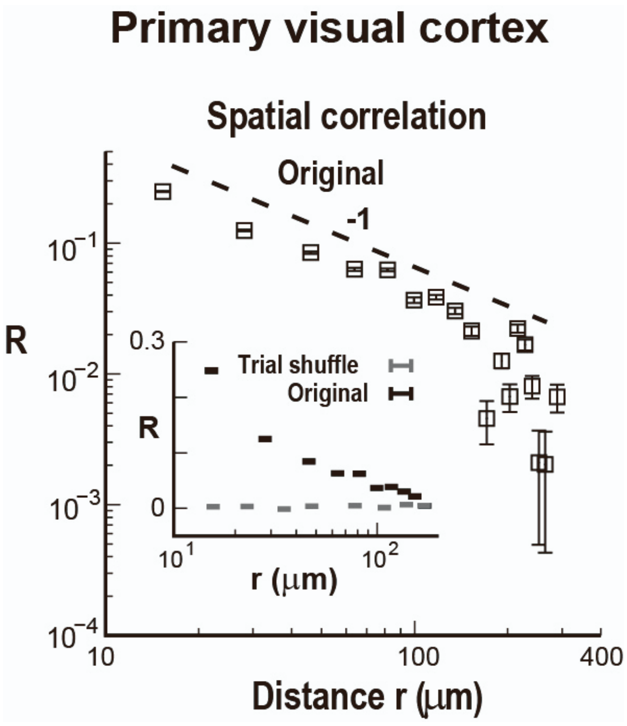

Figure S2 Pearson's pairwise correlations decay with distance approximately as a power law

Power-law decay of the average correlation as function of distance for the 2PI V1 dataset as function of distance. *Inset*: Trial-shuffling destroys correlations.

Supplemental Information
Figure S3

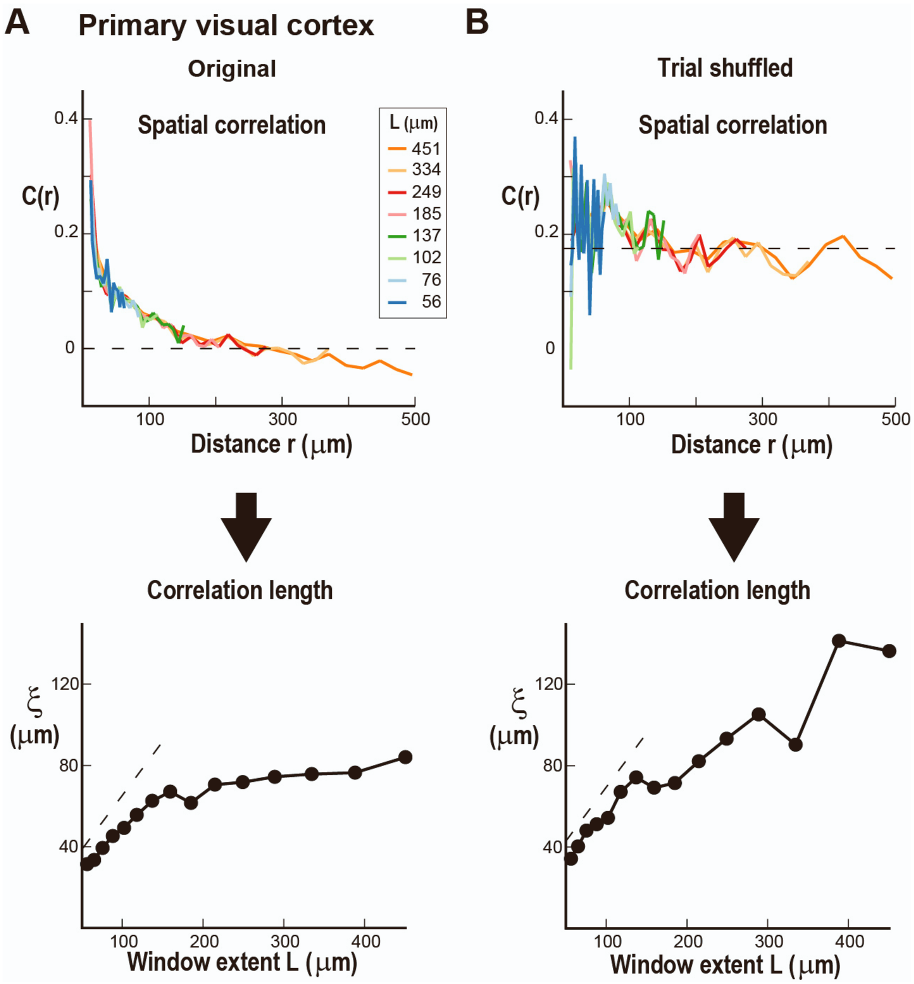

Figure S3 Linear growth of the correlation length is insensitive to trial-by-trial interactions without population average subtraction for the V1 dataset

(A) *Top*: Pearson pair-wise correlations, without the population average subtraction, decay with distance between neurons. Averages for *n* = 7 mice. *Colors*: window length. *Broken line*: zero correlation. *Bottom*: Correlation length grows linearly with the size of the window of observation only up to ~150 μm. (B) Same analysis as in *A*, but after performing trial-shuffling on the data. Note that the correlation length grows similarly to the original data with the window extent *L*.

Supplemental Information
Figure S4

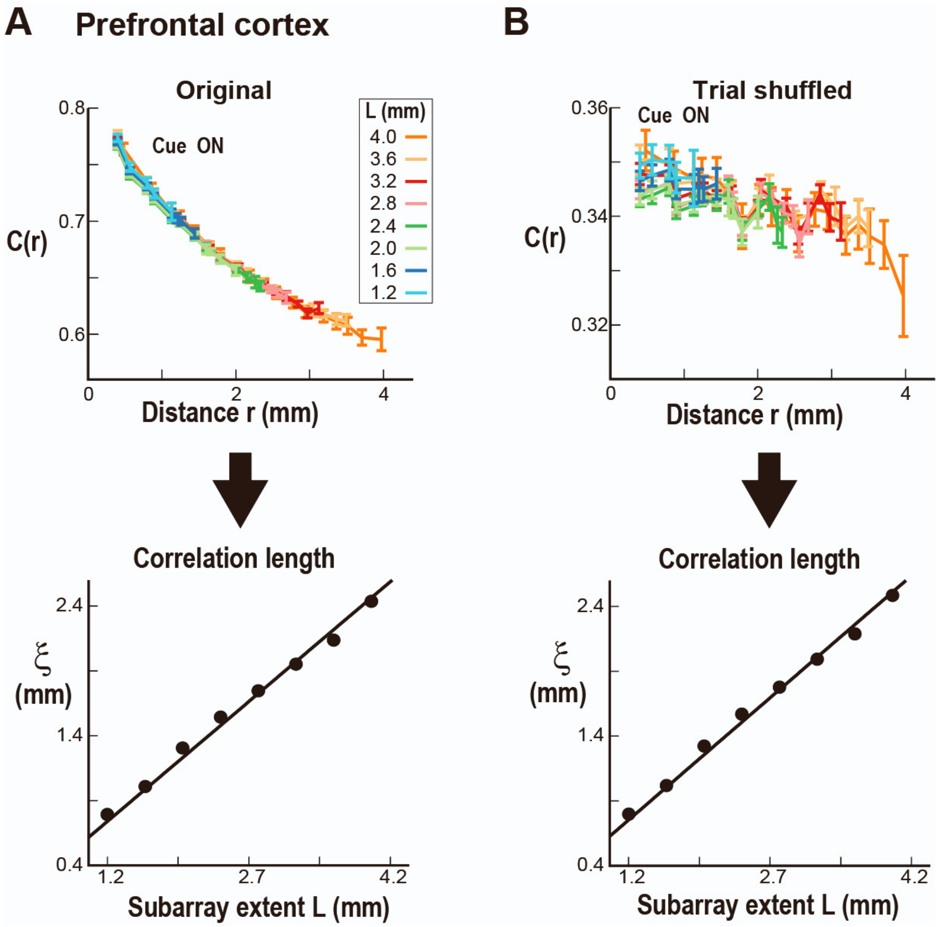

Figure S4 Linear growth of the correlation length is insensitive to trial-by-trial interactions when common input is not removed for the prefrontal dataset

(A) *Top*: Non-collapsed average Pearson correlation as function of distance for different subarrays of side length *L* (*color code*). Prefrontal cortex (monkey B). *Bottom*: Correlation length (obtained by the integral method) grows linearly with subarray extent. (B) Same as *A*, but for trial-shuffled data. Note that correlations remain positive despite destroying intra-trial interactions (*top*), leading to the same linear growth for the respective correlation length (*bottom*). For the linear regressions in *A* and *B*, chi-square test $p < 10^{-4}$.
